# The role of BHLHE40 in clinical features and prognosis value of PDAC by comprehensive analysis and *in vitro* validation

**DOI:** 10.3389/fonc.2023.1151321

**Published:** 2023-06-12

**Authors:** Chao Liu, Jiang Du, Jianwei Zheng, Ruizhe Zhang, Jialin Zhu, Bofan Xing, Lin Dong, Qianqian Zhou, Xiaofeng Yao, Song Gao, Yu Wang, Yu Ren, Xuan Zhou

**Affiliations:** ^1^ Department of Maxillofacial and Otorhinolaryngological Oncology, Tianjin Medical University Cancer Institute and Hospital, Key Laboratory of Cancer Prevention and Therapy, Tianjin Cancer Institute, National Clinical Research Center of Cancer; Tianjin’s Clinical Research Center for Cancer, Tianjin, China; ^2^ Department of Diagnostic and Therapeutic Ultrasonography, Tianjin Medical University Cancer Institute and Hospital, Key Laboratory of Cancer Prevention and Therapy, Tianjin Cancer Institute, National Clinical Research Center of Cancer, Tianjin’s Clinical Research Center for Cancer, Tianjin, China; ^3^ Department of Pancreatic Cancer, Tianjin Medical University Cancer Institute and Hospital, Key Laboratory of Cancer Prevention and Therapy, Tianjin Cancer Institute, National Clinical Research Center for Cancer, Tianjin’s Clinical Research Center for Cancer, Tianjin, China; ^4^ Department of Genetics, School of Basic Medical Sciences, Tianjin Medical University, Tianjin, China

**Keywords:** BHLHE40, PDAC, prognosis, risk model, tumor microenvironment

## Abstract

Pancreatic ductal adenocarcinoma (PDAC) is the leading cause of cancer-related mortality, primarily due to the abundance of cancer-associated fibroblasts (CAFs), depleted effector T cells, and increased tumor cell stemness; hence, there is an urgent need for efficient biomarkers with prognostic and therapeutic potential. Here, we identified BHLHE40 as a promising target for PDAC through comprehensive analysis and weighted gene coexpression network analysis of RNA sequencing data and public databases, taking into account the unique characteristics of PDAC such as cancer-associated fibroblasts, infiltration of effector T cells, and tumor cell stemness. Additionally, we developed a prognostic risk model based on BHLHE40 and three other candidate genes (ITGA2, ITGA3, and ADAM9) to predict outcomes in PDAC patients. Furthermore, we found that the overexpression of BHLHE40 was significantly associated with T stage, lymph node metastasis, and American Joint Committee on Cancer (AJCC) stage in a cohort of 61 PDAC patients. Moreover, elevated expression levels of BHLHE40 were validated to promote epithelial–mesenchymal transition (EMT) and stemness-related proteins in BXPC3 cell lines. Compared to the parent cells, BXPC3 cells with BHLHE40 overexpression showed resistance to anti-tumor immunity when co-cultured with CD8^+^ T cells. In summary, these findings suggest that BHLHE40 is a highly effective biomarker for predicting prognosis in PDAC and holds great promise as a target for cancer therapy.

## 1 Introduction

Pancreatic ductal adenocarcinoma (PDAC) is the seventh most deadly malignancy in the world (1) and the fourth and sixth most common cause of cancer-related mortality in the USA and China, respectively. It is predicted to become the second leading cause in the USA by 2030 (2). Although survival rates for other cancer types have been increasing steadily, the survival rate of PDAC has decreased slightly, resulting in a median overall survival time of no more than 6 months and a 5-year survival rate of 8% (3, 4). Of PDAC patients, 50% have already developed tumor metastasis at the time of diagnosis, and only 10%–15% of patients have the opportunity to receive surgical treatment (3). Low rates of diagnosis at early stages, rapid tumor progression, and disappointing surgical resection margins are the main reasons for the poor prognoses and high mortality associated with PDAC. There is thus an urgent need to develop efficient strategies to improve the rate of early PDAC diagnosis and better predict PDAC progression.

Aberrant gene expression and mutation are substantial causes of cancer progression and negative prognoses in patients with PDAC (5). Ninety percent of patients with pancreatic cancer exhibit KRAS mutation, and KRAS is one of the most important driver genes for pancreatic cancer development. In one study, approximately 50%–80% of patients had inactivation mutations in SMAD4, CDKN2A, and TP53 genes (6). In addition to these core mutations, there are many undiscovered genes involved in pancreatic cancer development. It is thus important to better understand the tumorigenesis of PDAC at the genomics level and identify novel biomarkers that can facilitate early diagnosis of PDAC and serve as efficient therapeutic targets.

Tumor immune microenvironments (TIMEs) are complex and constantly changing. They contain stromal cells, endothelial cells, and fibroblasts, and they are also affected by the precise regulation of various immune cells (7). Typical characteristics of the TIME in PDAC include severe interstitial fibrosis and highly immunosuppressive responses, resulting in PDAC’s highly aggressive nature and tendency to readily progress to metastatic malignancy (8). Cancer-associated fibroblasts (CAFs) are an important component of the TIME, and in PDAC, they can induce stromal fibrous tissue hyperplasia resulting in disease progression and drug resistance (9).

As well as large amounts of CAFs, regulatory T cells, pancreatic stellate cells (PSCs), myeloid cells, and cancer stem cells are all involved in PDAC invasion and metastasis (8, 10–12). Various recent studies have constructed prognostic models via the use of bioinformatics analysis to search for markers that affect the diagnosis and prognosis of pancreatic cancer (13–15). These studies have mainly focused on immunological and prognostic analyses rather than biomarker screening, however; thus, there is a need to identify biological PDAC TIME molecules with diagnostic and therapeutic values.

BHLHE40 is a member of the helix-loop-helix transcription factor family, which facilitates protein–protein interactions via its HLH domain and subsequently binds to target gene promoters to regulate transcription. Notably, BHLHE40 plays a pivotal role in the pathogenesis of colorectal cancer, breast cancer, and gastric cancer (16–18). BHLHE40 can be activated by various signals or stimuli, including hypoxia, immune factors, growth factors, and oxidative stress. These activations are involved in the processes of tumor proliferation, differentiation, and immunity (19). However, limited studies have been conducted on the role of BHLHE40 in PDAC. In this study, we aimed to investigate the crucial function of BHLHE40 in PDAC progression. Through comprehensive analysis of RNA sequencing and public databases, we identified BHLHE40 as a valuable biomarker associated with CAFs, immune cell infiltration, and tumor cell stemness in PDAC. Meanwhile, a risk model was constructed utilizing BHLHE40, ITGA2, ITGA3, and ADAM9 to prognosticate patient survival in PDAC. *In vitro* experiments demonstrated that overexpression of BHLHE40 promoted tumor cell invasion and stemness while inhibiting anti-tumor immunity by inducing apoptosis of CD8^+^ T cells. Furthermore, clinical samples revealed a significant correlation between BHLHE40 expression levels and PDAC prognosis and progression. Collectively, these findings suggest that BHLHE40 represents a promising therapeutic target for the treatment of PDAC tumorigenesis and progression.

## 2 Materials and methods

### 2.1 Data collection

Fresh tumor samples were collected from 61 patients from Tianjin Medical University Cancer Hospital. All patients were approved by the ethics committee and provided signed informed consent. The transcriptome sequencing data and clinical information of pancreatic adenocarcinoma in The Cancer Genome Atlas (TCGA) were downloaded from the XENA database (https://xenabrowser.net/). Transcriptome data for single-cell sequencing of the CRA001160 dataset were downloaded from the Genome Sequence Archive (GSA) database.

### 2.2 RNA sequencing

In our study, RNA sequencing was performed using the TruSeq RNA Library Preparation Kit, which is one of the RNA library preparation kits from Illumina. This kit is designed to prepare libraries from RNA samples and is suitable for mRNA, miRNA, total RNA, and full-length transcript sequencing applications. The specific protocol is as follows: 1) mRNA isolation: poly-T oligo(dT) magnetic beads are used to isolate mRNA molecules from total RNA samples. 2) RNA fragmentation: mRNA molecules are randomly fragmented into numerous short fragments using chemical or mechanical methods. 3) cDNA synthesis: RNA fragments are reverse-transcribed into double-stranded cDNA in the RNA–DNA hybrid form. 4) End repair and A-tailing: T4 DNA polymerase is used to add an A base to the cDNA ends, followed by end repair to ensure the integrity of the ends and compatibility with Illumina adapter structures. 5) Adapter ligation: Illumina adapter sequences are ligated to the library cDNA. 6) PCR amplification: the library cDNA is PCR-amplified to obtain sufficient DNA yield. 7) Size selection and quantification: appropriate-sized libraries are selected using gel electrophoresis or other methods, and the DNA concentration of the libraries is measured using a fluorometer or other methods. Illumina HiSeq 2000 sequencer was used for double-ended sequencing. After quality control of the base quality and nucleotide composition of the sequence, the STAR algorithm was used to align the human reference genome (Hg38). Samtools was applied to sequence and remove repeated sequences from reads; HTseq calculated the RNA expression levels of each gene to conduct subsequent analysis (**Supplementary Table 1**).

### 2.3 Single-cell sequencing analysis

The single-cell transcriptome files downloaded from the GSA database were dimensionally reduced for clustering by using the R package “Seurat”. Unsupervised clustering analysis was performed with Uniform Manifold Approximation and Projection (UMAP) method. The R package “singleR” and characteristic markers were used to identify the cell subsets.

### 2.4 Weighted gene coexpression network analysis

Weighted gene coexpression network analysis (WGCNA) is an effective method for obtaining the expression patterns of multiple genes in different samples in order to identify the genes with similar expression patterns (20). The characteristic genes of different modules were screened according to clinical features, stromal score, immune score, and stemness index in TCGA. Soft-threshold β = 7 and scale-free R^2 ^= 0.880 were selected as the threshold. To evaluate the interactions between networks, the weighted adjacency matrix was transformed into the topological overlap matrix (TOM). According to the corresponding clinical features and scores of each module, the genes were classified into different modules, and Pearson’s correlation method was performed to evaluate the correlation. The genes in three core modules were finally selected for further screening using the Venn diagram.

### 2.5 Differential analysis and enrichment analysis

Differentially expressed genes (DEGs) were screened by the R package “limma”, and *p* < 0.05 and |Log_2_FC| > 1 were isolated to further analyze the function. Gene Ontology (GO) and Kyoto Encyclopedia of Genes and Genomes (KEGG) enrichment analyses were performed to explore the function of DEGs. Moreover, gene set enrichment analysis (GSEA) was performed to further verify their functions. The *p*-value cutoff was 0.01, and the minimum enrichment factor was 1.5, showing the top 2 pathways to visualize by R package “ggplot2”. In this study, the clusterProfiler package and xiantao platform were utilized for KEGG pathway analysis and visualization. The clusterProfiler package is an R package that can be used for enrichment analysis and visualization of GO, KEGG, and Reactome pathways. The xiantao platform is mainly used for the visualization of clusterProfiler package analysis results and can be accessed at https://www.xiantao.love/.

### 2.6 Model construction and verification

The R package “glmnet” was used for least absolute shrinkage and selection operator (LASSO) regression analysis using 10-fold cross-validation to determine the best λ value in the regression model, and to evaluate the risk factors of the selected genes, Cox regression analysis was performed. Nomogram was developed by the R package “rms” to visualize the prognosis model. Finally, the Kaplan–Meier (K-M) analysis and receiver operating characteristic (ROC) curve analysis were conducted for verifying the accuracy and reliability of the risk model.

### 2.7 Immune infiltration analysis

ESTIMATE is an evaluation method that could predict the stromal cell proportion and the extent of immune infiltration. The higher the stromal score, the higher the proportion of stromal cells predicted. The higher the immune score, the more abundant the immune infiltration predicted. The CIBERSORT algorithm assessed differences in the proportion of cell types between patients. The tumor stemness index and the proportion of immune cells were calculated by single-sample GSEA (ssGSEA), which is a widely used algorithm to evaluate the composition and proportion of different immune cell types in each sample (21). We finally determined 24 immune cell types and compared the differences in immune cell enrichment between different groups.

### 2.8 Western blotting

Cells were lysed in lysis buffer for 30 min and centrifuged at 4°C for 15 min; then, the supernatant was collected and stored until use. Pierce™ BCA protein detection kit (Thermo, Waltham, MA, USA) was used to determine the protein concentration in the supernatant. After an appropriate amount of loading buffer was added, the proteins were denatured at high temperatures, and then sodium dodecyl sulfate–polyacrylamide gel electrophoresis (SDS-PAGE) was performed. Proteins were transferred from gel to the polyvinylidene difluoride (PVDF) membrane. Bovine serum albumin or 5% skimmed milk was used to block the non-specific binding sites. The membrane were incubated overnight at 4°C with corresponding specific antibodies: GAPDH (Proteintech, Chicago, IL, USA; 60004-1-Ig), BHLHE40 (Proteintech, 17895-1-AP), Snail (CST, Danvers, MA, USA; 3879T), Slug (CST, 9585T), Vimentin (CST, 5741T), ZEB1 (CST, 3396T), SOX9 (Abcam, Cambridge, UK; ab185966), Oct4 (Abcam, ab19857), and CD133 (Abcam, ab19898). Then, the second antibody was incubated for 1 h at room temperature, and the enhanced chemiluminescence (ECL) method was used to detect the expression of related proteins.

### 2.9 Establishment of stable cell lines

We constructed a pCDH plasmid containing the CDS sequence of human BHLHE40 and used pCDH vector as control. Then, we performed lentiviral infections according to standard procedures and used puromycin (Solarbio, Beijing, China) to screen stable cells.

### 2.10 RT-PCR

TRIzol reagent (Solarbio) was used to extract the total RNA from cells, and then RNA was transcribed into cDNA using PrimeScript™ RT Reagent Kit (Takara, Mountain View, CA, USA). The TB Green® Premix Ex Taq™ II Kit was used according to the manufacturer’s instructions, and ABI QuantStudio5 was used to detect real-time fluorescence.

### 2.11 Wound-healing assay

Cells were seeded into six-well plates and cultured until the fusion degree reached 90%. The plates were scraped with 10-μl tip and washed with phosphate-buffered saline (PBS); then, the cells were incubated at 37°C with serum-free culture medium. Three random fields were photographed using a microscope at appropriate time points.

### 2.12 Cell migration and invasion assay

A total of 1 × 10^5^ cells in 100 µl serum-free culture medium were added into the upper layer of transwell uncoated or coated with Matrigel (BD Biosciences), and 600 µl culture medium containing 10% serum was added in the lower chamber. After 24 h, the migrating cells were fixed with 4% paraformaldehyde and stained with crystal violet. Finally, three random fields were selected under the microscope and counted.

### 2.13 Sphere formation assay

A total of 5 × 10^3^ cells were seeded in low-adhesion plates in serum-free culture medium, which contained 2% B27 (Invitrogen, Carlsbad, CA, USA), EGF (Proteintech, 20 ng/ml), FGF2 (Proteintech, 10 ng/ml), insulin (Sigma, St. Louis, MO, USA; 5 μg/ml), and 0.4% bovine serum albumin (Sigma). After 2 weeks, tumor spheres with a diameter >75 μm were counted.

### 2.14 Flow cytometry

CD8^+^ T cells were purified from healthy human peripheral blood mononuclear cells using CD8^+^ T Cell Isolation Kit (Miltenyi Biotec, Bergisch Gladbach, Germany) according to the instructions of the manufacturer. Purified CD8^+^ T cells were cultured in a medium containing anti-CD3 and anti-CD28 for 72 h and then used for further experiments. A total of 1 × 10^5^ CD8^+^ T cells were co-cultured with 5 × 10^5^ tumor cells for 72 h. The apoptosis and phenotype of CD8^+^ T cells were analyzed. The data were analyzed using FlowJo10.0.

### 2.15 Immunohistochemical staining

The paraffin-embedded tissue sections were dried overnight at 70°C. Then, the slices were soaked in dimethylbenzene and ethanol with different concentration gradients. The slices were washed three times, and then heat-mediated antigen repair was performed. After the antigen repair solution was cooled, the sections were treated according to the instructions of the Two-Step Method Kit (ZSGB Bio, Beijing, China), and the anti-BHLHE40 (Proteintech, 17895-1-AP, 1:200) antibody was added to the sections and incubated at 4°C overnight. The next day, added horseradish peroxidase (HRP)-labeled reagent II to the slice. Then, DAB staining and hematoxylin staining were performed. After dehydration and drying, neutral gum was used as sealant. The immunohistochemical score was performed by two independent and experienced pathologists.

### 2.16 Statistical analysis

In order to compare the difference between the different groups, the Wilcoxon rank sum test was used for continuous variables. *p* < 0.05 was considered statistically significant. All the analyses were based on R language software (version 4.1.0). *, *p*<0.05; **, *p*<0.01; ***, *p*<0.001; ****, *p*<0.0001.

## 3 Results

### 3.1 CAFs, effector T-cell infiltration, and tumor cell stemness are closely associated with PDAC development

**Figure 1:**
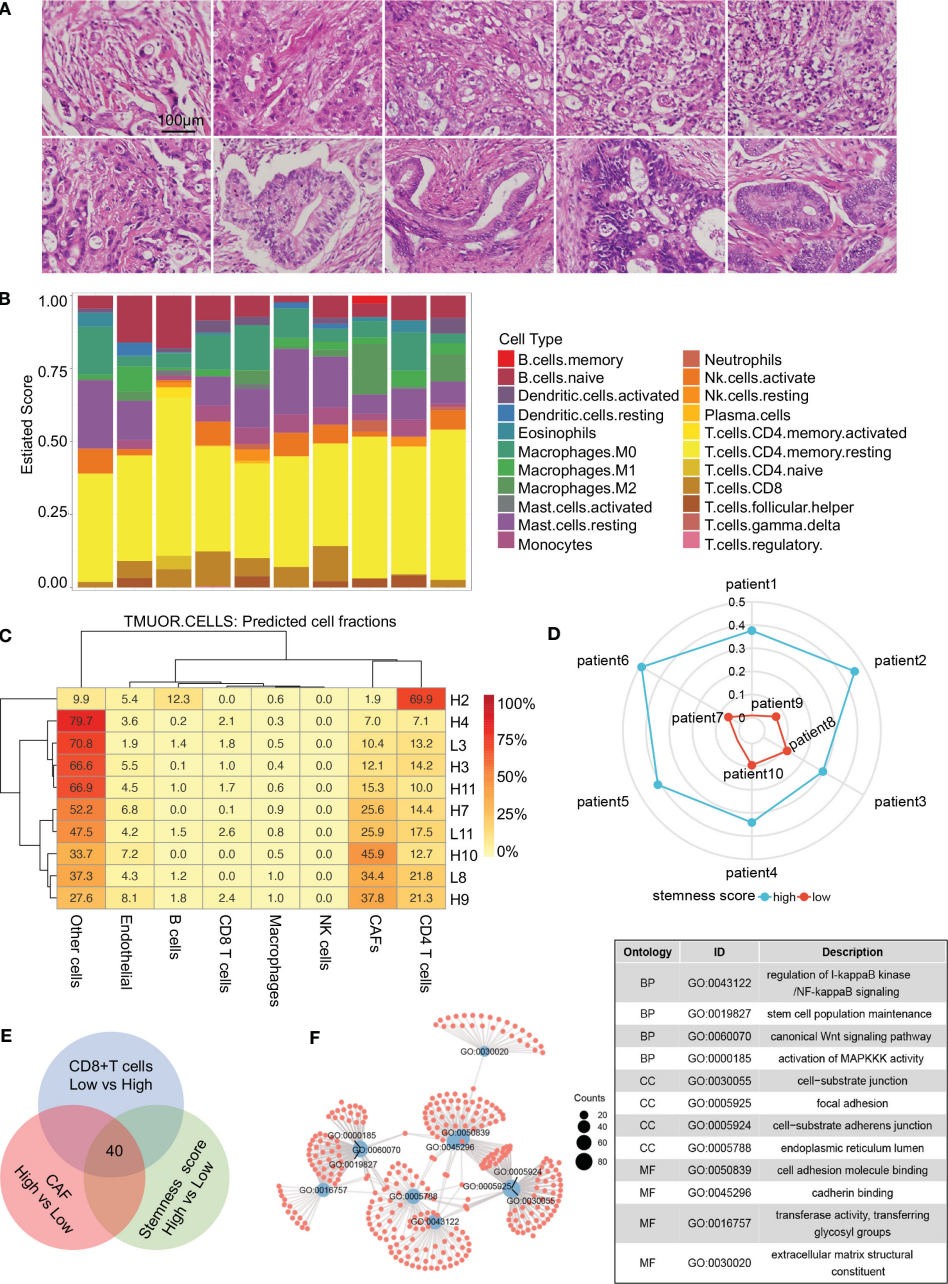
Screening process for core genes. **(A)** HE staining of 10 patients with RNA sequencing. **(B)** CIBERSORT revealing the tumor microenvironment of 10 patients. **(C)** Proportions of cells in different patients. **(D)** Radar diagram depicting the stemness index of 10 patients. **(E)** Venn diagram showing 40 genes obtained by the intersection of CAF, CD8^+^ T-cell proportion, and stemness index. **(F)** Gene Ontology pathway intersections. CAF, cancer-associated fibroblast.

The typical characteristics of the pancreatic cancer microenvironment are severe interstitial fibrosis, immunosuppression, and aggressive tumor cells. Large numbers of CAFs, exhausted effector T cells, and increased tumor cell stemness are associated with the promotion of PDAC occurrence and development. Transcriptome sequencing was performed on 10 PDAC samples (**Supplementary Table 1**), and tissue sections of the same samples were stained with hematoxylin and eosin (HE) to evaluate tumor differentiation (**Figure 1A**). Infiltration of different immune cells was then analyzed in all patients via CIBERSORT software (**Figure 1B**). The 10 patients were divided into two groups based on the CD8^+^ T-cell infiltration ratio. Differential gene expression analysis was performed, and 1,021 highly expressed genes were identified in the low CD8^+^ T-cell infiltration group. Interstitial fibrosis was then evaluated in the 10 patients (**Figure 1C**). Differential gene expression analysis based on the proportion of CAFs was performed, and 942 DEGs were identified in the high fibrosis group. A stemness index was used to evaluate stemness level (**Figure 1D**), and 784 DEGs were identified in patients with high stemness indexes. DEGs in the low CD8^+^ T-cell infiltration/high fibrosis/high stemness index group were selected for intersection display via a Venn diagram, and 40 potential genes were selected (**Figure 1E**). GO pathway enrichment analysis was performed to evaluate the functions of DEGs (**Figure 1F**). Enriched DEGs were mainly involved in NF-κB signaling, stem cell population maintenance, Wnt signaling, MAPK signaling, cell substrate junction, focal adhesion, extracellular matrix structural constituents, and cell adhesion molecule binding, all of which were closely associated with PDAC progression. These results revealed that increased proportions of CAFs, decreased CD8^+^ T-cell infiltration, and increased tumor cell stemness are crucial factors that affect the development of PDAC.

### 3.2 BHLHE40, ITGA3, ITGA2, and ADAM9 are target genes associatedwith PDAC development

**Figure 2:**
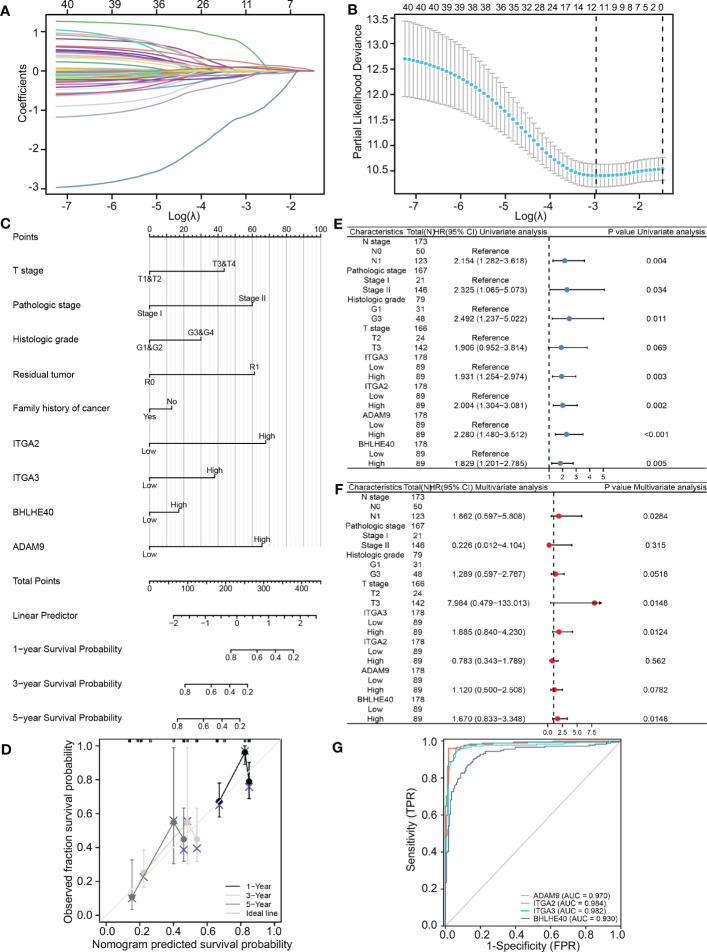
Construction and validation of the LASSO regression model. **(A)** The determination of four genes according to LASSO regression analysis. **(B)** Lambda curves showing cross-validation in LASSO analysis. **(C)** Nomogram showing clinical features and effects of four variables on prognosis. **(D)** Calibration curves indicating that the nomogram accurately predicts survival. **(E)** Univariate Cox analysis of different clinical characteristics and genes. **(F)** Multivariate Cox analysis of different clinical characteristics and genes. **(G)** ROC curves using ITGA2, ITGA3, ADAM9, and BHLHE40 categorical variables indicating that the four genes had reliable predictive accuracy. LASSO, least absolute shrinkage and selection operator; ROC, receiver operating characteristic.

TCGA database was used to further identify important biomarkers derived from 40 DEGs. Four were screened out via LASSO regression and matching λ values—ITGA3, ITGA2, BHLHE40, and ADAM9 (**Figures 2A, B**)—which collectively make the largest contribution to prognosis and survival in PDAC patients. A nomogram was constructed based on clinical parameters, and four variables were used to predict the survival rate of patients with pancreatic cancer. These four selected variables had favorable prediction efficiency (**Figure 2C**), and a calibration curve was used to further calibrate the construction of the model (**Figure 2D**). Univariate and multivariate analyses of the four biomarkers and clinical parameters were then performed. In univariate Cox regression analysis, the hazard ratio (HR) coefficients of the four parameters were all >1.0 (ITGA2 HR 2.004, ITGA3 HR 1.931, ADAM9 HR 2.280, and BHLHE40 HR 1.829) (**Figure 2E**). In the multivariate analysis, ITGA3 (HR 1.885, *p* = 0.0124) and BHLHE40 (HR 1.670, *p* = 0.0148) were statistically significant predictors of aggressive PDAC progression (**Figure 2F**). Next, combined with the roles of the four biomarkers, ITGA2, ITGA3, ADAM9, and BHLHE40 were used as categorical variables to generate ROC curves, identifying benign and malignant pancreatic tissues. The areas under the curve (AUCs) were 0.970 for ADAM9, 0.984 for ITGA2, 0.982 for ITGA3, and 0.930 for BHLHE40, indicating the important role of ITGA2, ITGA3, ADAM9, and BHLHE40 in the prognosis of PDAC (**Figure 2G**). ITGA2, ITGA3, ADAM9, and BHLHE40 expressions were negatively associated with overall survival and disease-free survival (**Supplementary Figure 1**), according to TCGA database. These results suggested that BHLHE40, ITGA2, ITGA3, and ADAM9 were strongly associated with the development of PDAC.

### 3.3 BHLHE40, ITGA3, ITGA2, and ADAM9 are informative predictors of prognosis and clinical outcome in PDAC

**Figure 3:**
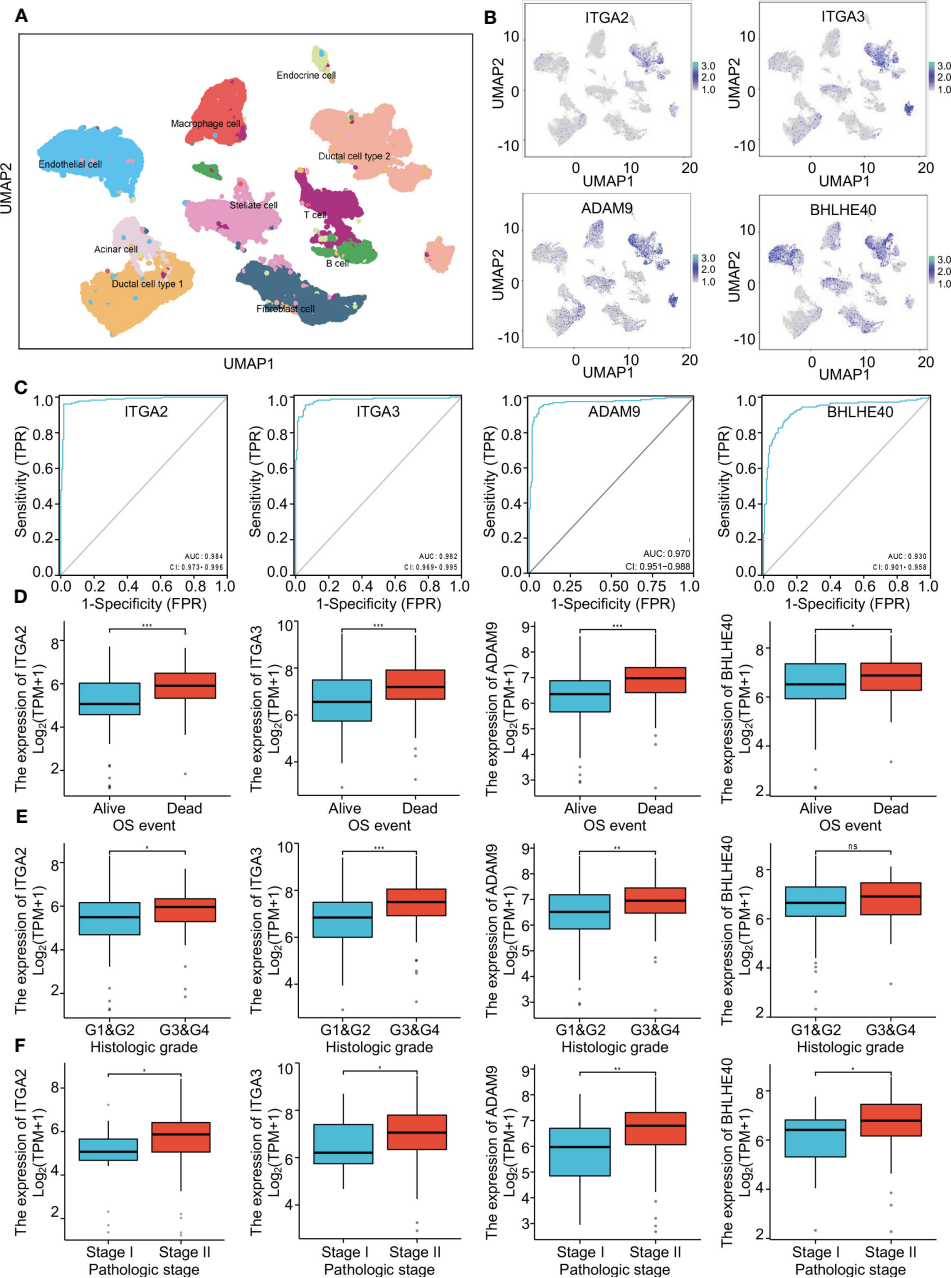
The critical role of four genes in pancreatic cancer. **(A)** Single-cell analysis of the pancreatic cancer tumor microenvironment. **(B)** The distributions of ITGA2, ITGA3, ADAM9, and BHLHE40 in different cell populations. **(C)** ROC curves using ITGA2, ITGA3, ADAM9, and BHLHE40 as categorical variables confirmed that the four genes had great diagnostic value in PDAC. **(D–F)** Associations between ITGA2, ITGA3, ADAM9, and BHLHE40 and OS event, histological grade, and pathological stage. ROC, receiver operating characteristic; PDAC, pancreatic ductal adenocarcinoma; OS, overall survival.

**Figure 4:**
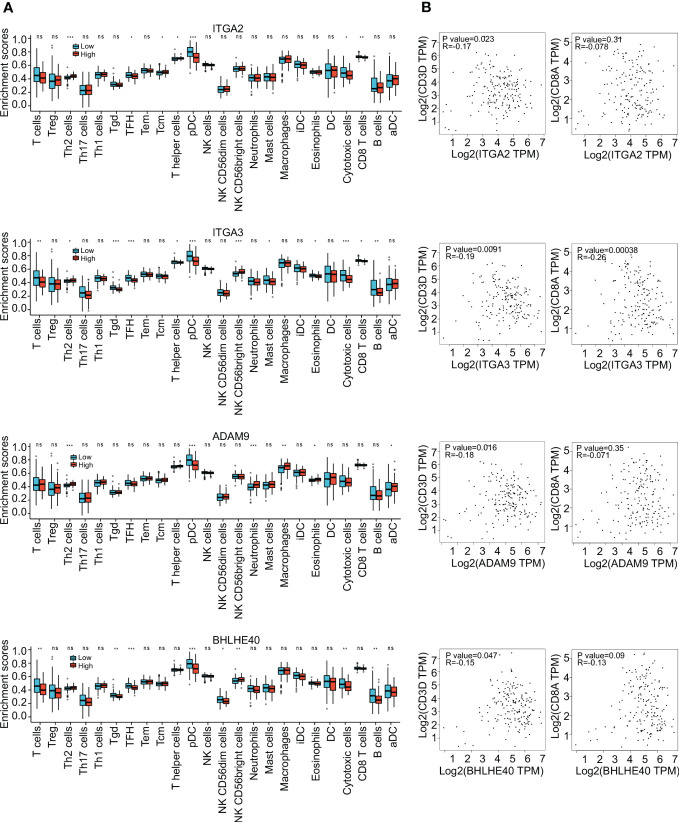
Immune infiltration analysis. **(A)** Enrichment scores of different immune cells in groups with high and low expression of ITGA2, ITGA3, ADAM9, and BHLHE40. **(B)** Correlations between expression of ITGA2, ITGA3, ADAM9, and BHLHE40. *p<0.05; **p<0.01; ***p<0.001; ns, no significance in statistics.

To further investigate the clinical benefit of the four selected variables during PDAC treatment, single-cell transcriptome PDAC data were downloaded from the GSA database (CRA001160 dataset) for analysis. Unsupervised clustering analysis was performed with the UMAP method, and R studio software (Seurat package) was used to identify cell clusters. A ductal cell subgroup was identified via analysis of KRT19 and EPCAM, and MUC1 and FXYD3 were used to further characterize malignant ductal cells (**Supplementary Figure 2A**). Lastly, ductal cell 2 cluster was associated with malignant ductal cells, ductal cell 1 cluster was associated with normal ductal cells, and the aforementioned four biomarkers were highly expressed in ductal cell 2 cluster (**Figures 3A, B**, **Supplementary Figure 2B**). In order to confirm the diagnostic value of four selected genes, ITGA2, ITGA3, ADAM9, and BHLHE40 were used as categorical variables to generate ROC, and AUCs were 0.984 for ITGA2, 0.982 for ITGA3, 0.970 for ADAM9, and 0.930 for BHLHE40 (**Figure 3C**). High expression levels of ITGA2, ITGA3, ADAM9, and BHLHE40 were significantly associated with shorter overall survival, higher differential grade, higher pathological stage, and worse therapy outcome (**Figures 3D–F**, **Supplementary Figure 3**). Associations between the four target genes and immune cell infiltration were assessed. Expression levels of ITGA2, ITGA3, ADAM9, and BHLHE40 were negatively associated with the infiltration of many kinds of immune cells, as well as CD3D mRNA and CD8A mRNA expression (**Figures 4A, B**). These results indicated that the biomarkers ITGA2, ITGA3, ADAM9, and BHLHE40 were potentially informative with respect to PDAC diagnosis and prognostic predictions at both the molecular level and the clinical level.

### 3.4 Risk model based on coexpression of ITGA3, ITGA2, BHLHE40, and ADAM9 predicts PDAC prognosis

To investigate the prediction of PDAC tumor-promoting characteristics and survival probability based on ITGA2, ITGA3, ADAM9, and BHLHE40, LASSO regression analysis was used to screen for coexpression, and the results were used to construct a risk model. The risk score was derived via the following formula:


Risk score = 0.8 × (ITGA2) + 1.2 × (ITGA3) + 2.3 × (ADAM9) + 1.4 × (BHLHE40)

**Figure 5:**
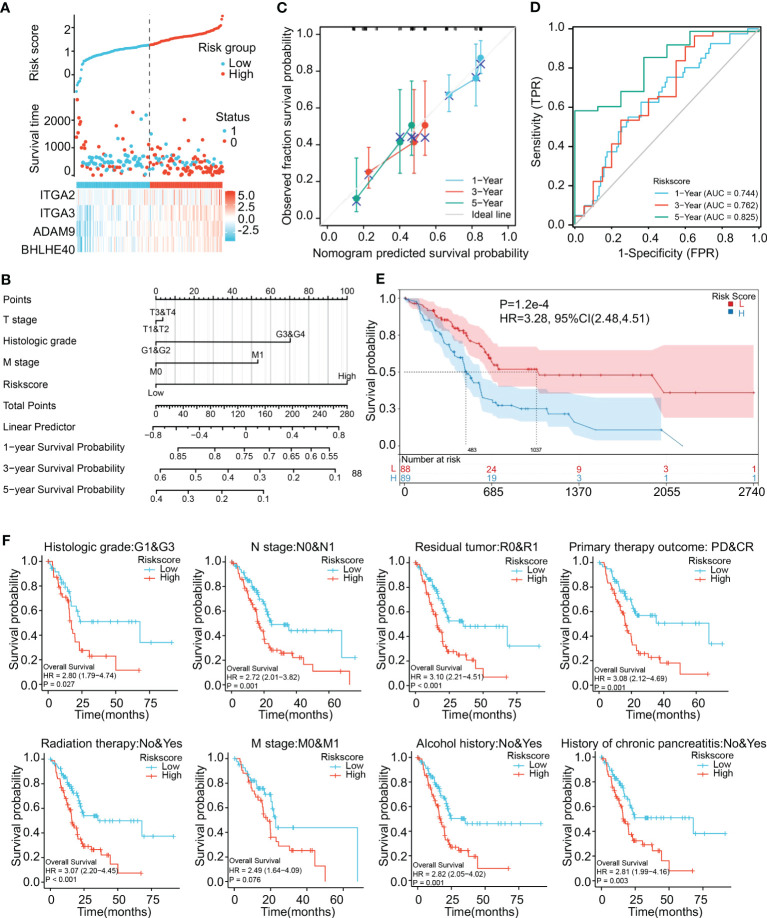
Construction and validation of a risk model. **(A)** Construction of a risk model based on ITGA2, ITGA3, ADAM9, and BHLHE40 expression showing prediction of survival. **(B)** Nomogram showing clinical features and the effects of risk score on prognosis. **(C)** Calibration curves using the risk score as a categorical variable and overall survival as a continuous variable indicating that the nomogram accurately predicts survival. **(D)** ROC curves using the risk score as a categorical variable indicating that risk score exhibits reliable predictive accuracy. **(E)** K-M survival analysis indicating that risk score was significantly associated with prognosis. **(F)** Risk scores affecting the prognoses of patients with differences in histological grade, N stage, residual tumor, primary treatment outcome, radiation therapy, M stage, alcohol history, and chronic pancreatitis history. ROC, receiver operating characteristic; K-M, Kaplan–Meier.

Risk scores thus derived were significantly associated with the prognosis of pancreatic cancer. Risk score was positively associated with expression levels of the four genes (**Figure 5A**). A nomogram incorporating risk score and clinical parameters was constructed, and it indicated that the predictive accuracy of the risk score in PDAC patients with respect to 1-, 3-, and 5-year overall survival was significantly higher than that of T stage, histologic grade, and M stage (**Figure 5B**). Calibration analysis in which the risk score was used as a categorical variable and overall survival as a continuous variable verified the validity and consistency of the model (**Figure 5C**). ROC analysis in which the risk score was used as a categorical variable suggested that the risk model had satisfactory predictive accuracy for 1-year (AUC 0.744), 3-year (AUC 0.762), and 5-year (AUC 0.825) survival (**Figure 5D**). In K-M curve analysis, the high-risk-score groups had significantly shorter median survival times (HR 3.28, *p* < 0.001) (**Figure 5E**). Parametric subgroup analysis was performed comparing the low-risk-score and high-risk-score groups. With regard to the predictive capacity of the risk score globally, the HR values were 2.80 for histological grade, 2.72 for N stage, 3.10 for residual tumor, and 3.08 for primary treatment outcome (**Figure 5F**). These results indicated that the risk model based on ITGA3, ITGA2, BHLHE40, and ADAM9 coexpression could effectively predict PDAC prognoses.

### 3.5 BHLHE40 is a critical prognostic gene in PDAC

**Figure 6:**
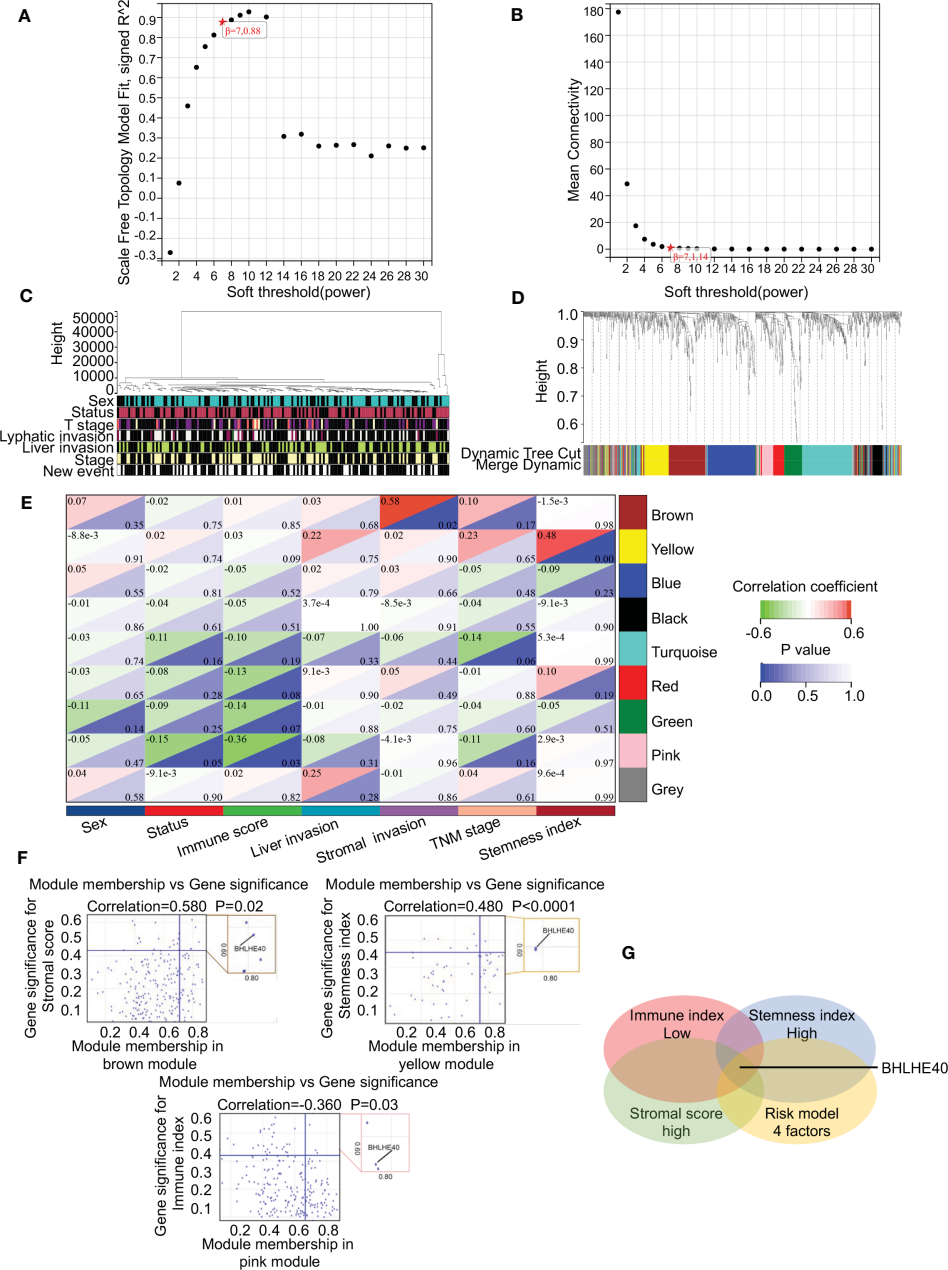
BHLHE40 was screened out by WGCNA. **(A, B)** WGCNA networks were constructed based on soft thresholds with β = 7 and scale-free R^2 = ^0.880. **(C)** Topological overlap clustering genes based on different characteristics. **(D)** Different colored cluster trees representing dissimilar gene sets. **(E)** Topological overlap matrix showing correlations between modules and different characteristics. **(F)** Scatter plots showing BHLHE40 by stromal score, stemness index, and immune index. **(G)** Venn diagram showing the intersections of the risk module with low immune index, high stromal score, and high tumor stemness index. WGCNA, weighted gene coexpression network analysis. ★, scale-free R^2^ and mean connectivity corresponding to soft-threshold powers (β).

WGCNA was performed to identify gene clusters with similar expression patterns and to investigate relationships between the modules and the characteristics of samples (**Figures 6A, B**). That analysis included 178 samples with associated clinical information (**Figure 6C**). After the removal of gray modules using a combined dynamic tree-cutting method, three coexpressed modules were identified (**Figure 6D**). There were 1,000 genes identified in the analysis that were mapped to the TOM, indicating that each module had been independently validated (**Figure 6E**). Brown, yellow, and pink modules containing BHLHE40 were correlated with stromal score (R^2 ^= 0.580, *p* = 0.02), stemness index (R^2 ^= 0.480, *p* < 0.001), and immune score (R^2^ = −0.360, *p* = 0.03). A scatter plot representing gene significance and module membership (colored modules and phenotypes) in pancreatic cancer patients is shown in **Figure 6F**. BHLHE40 was the only common gene between the weighted coexpressed network and the risk model (**Figure 6G**). BHLHE40 was considered to be one of the critical genes in these modules associated with poor PDAC prognosis.

### 3.6 BHLHE40 plays critical roles in PDAC tumorigenesis progression

**Figure 7:**
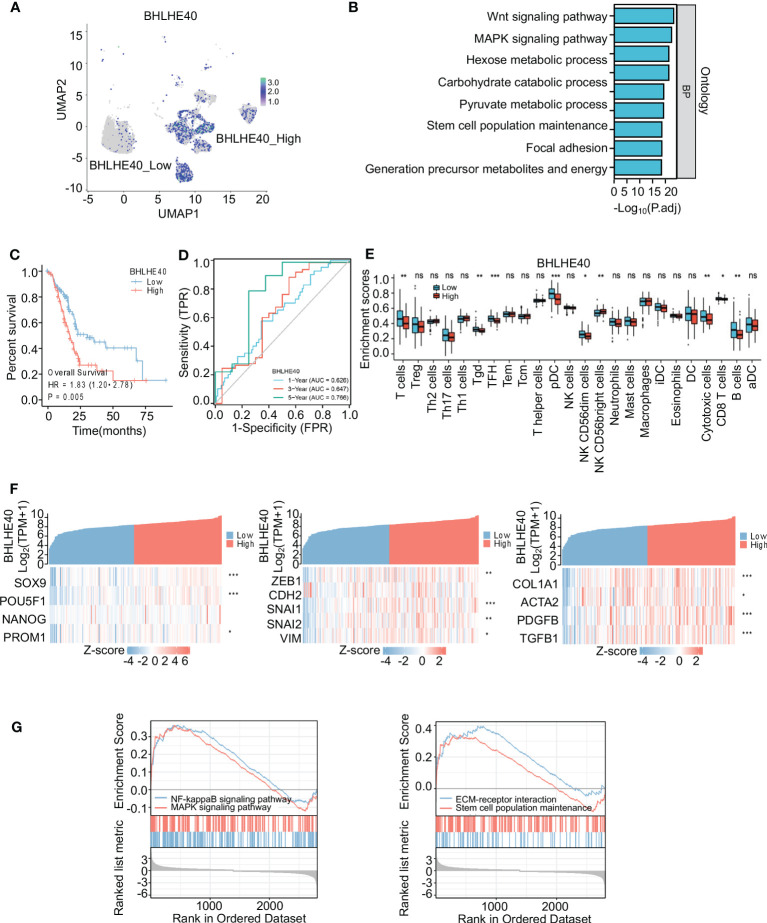
Identification and functional analysis of BHLHE40. **(A)** BHLHE40 expression in malignant ductal subsets. **(B)** Enrichment analysis of malignant ductal cells in high BHLHE40 and low BHLHE40 groups. **(C)** K-M survival curves in high and low BHLHE40 expression groups. **(D)** ROC curves indicating the accuracy of BHLHE40 for predicting survival. **(E)** Comparisons of enrichment scores of different immune cells in high and low BHLHE40 expression groups. **(F)** Coexpression heatmaps showing that BHLHE40 expression was associated with stemness-related genes, EMT-related genes, and PSC-related genes. **(G)** Gene set enrichment analysis of BHLHE40 expression. WGCNA, weighted gene coexpression network analysis; K-M, Kaplan–Meier; ROC, receiver operating characteristic; EMT, epithelial–mesenchymal transition; PSC, pancreatic stellate cell. *p<0.05; **p<0.01; ***p<0.001; ns, no significance in statistics.

The biological function of the BHLHE40 gene in PDAC was further investigated via single-cell sequencing. Malignant ductal cells were isolated, and subgroup analyses were conducted. Based on BHLHE40 colored-coded analysis and expression analysis, malignant epithelial cells were divided into the BHLHE40^high^ and BHLHE40^low^ subgroups (**Figure 7A**). Genes that were differentially expressed in these two subgroups were then analyzed, and KEGG enrichment analysis was performed. With regard to critical pathways, there were significant changes in genes related to cell population maintenance, the MAPK signaling pathway, the Wnt signaling pathway, and focal adhesion (**Figure 7B**). In analyses of TCGA database, BHLHE40 significantly predicted poor prognosis and lower survival time in patients with pancreatic cancer (HR 1.83, *p* = 0.005) (**Figure 7C**). In ROC analysis, BHLHE40 had considerable predictive accuracy with respect to survival rates in PDAC (1-year AUC 0.626, 3-year AUC 0.647, and 5-year AUC 0.766) (**Figure 7D**). In the comparative analysis of immune infiltration in patients in the BHLHE40^high^ and BHLHE40^low^ subgroups, high BHLHE40 expression was negatively correlated with levels of infiltration of T cells, γδT cells, follicular helper T cells, plasmacytoid dendritic cells, natural killer cells, cytotoxic cells, CD8^+^ T cells, and B cells (**Figure 7E**). Because BHLHE40 screening mainly considered the infiltration of immune cells, tumor cell stemness, and proportions of interstitial CAFs in the tumor microenvironment (TME), a coexpression heatmap was generated. In that analysis, BHLHE40 was significantly correlated with classical stemness genes (SOX9, POU5F1, and PROM1), epithelial–mesenchymal transition (EMT)-related genes (ZEB1, SNAI1, SNAI2, and VIM), and activated PSC-related genes (COL1A1, ACTA2, PDGFB, and TGFB1) (**Figure 7F**). Lastly, gene set enrichment analysis in the BHLHE40^high^ and BHLHE40^low^ subgroups indicated that high BHLHE40 expression was significantly associated with the enrichment of genes involved in stem cell population maintenance, the MAPK signaling pathway, the NF-κB signaling pathway, and the extracellular matrix (ECM)–receptor signaling pathway (**Figure 7G**).

**Figure 8:**
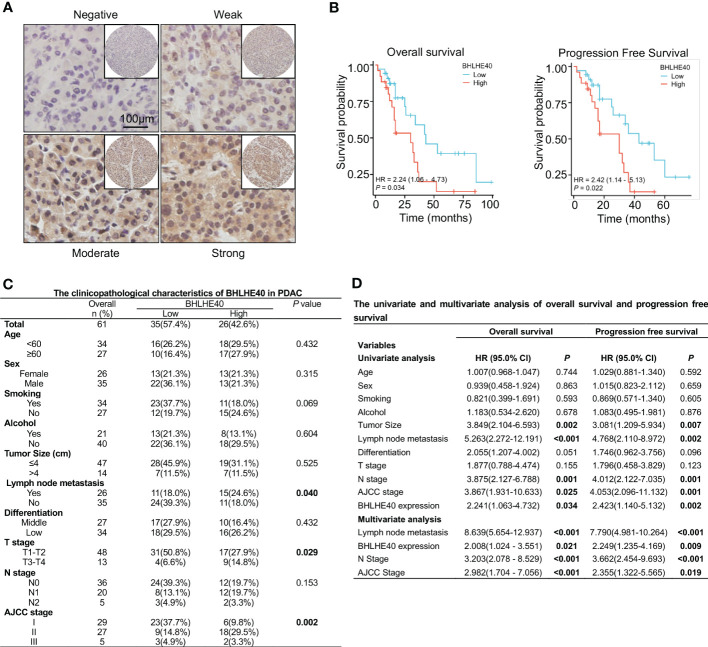
Clinical analysis of BHLHE40 in PDAC. **(A)** Representative immunohistochemistry staining of BHLHE40 expression. **(B)** Associations between BHLHE40 expression and overall survival and progression-free survival. **(C)** Clinicopathological characteristics of BHLHE40 in PDAC. **(D)** Univariate and multivariate analyses of BHLHE40 in PDAC. PDAC, pancreatic ductal adenocarcinoma.

The correlations between BHLHE40 expression and clinical characteristics of PDAC samples were assessed via immunohistochemical staining (**Figure 8A**). Compared with patients with low BHLHE40 expression, patients with high BHLHE40 expression had shorter overall survival and progression-free survival (**Figure 8B**). BHLHE40 expression was positively correlated with lymph node metastasis, T stage, and American Joint Committee on Cancer (AJCC) stage (**Figure 8C**). In univariate and multivariate analyses, lymph node metastasis, N stage, BHLHE40 expression, and AJCC stage were independently associated with poor prognosis (**Figure 8D**).

**Figure 9:**
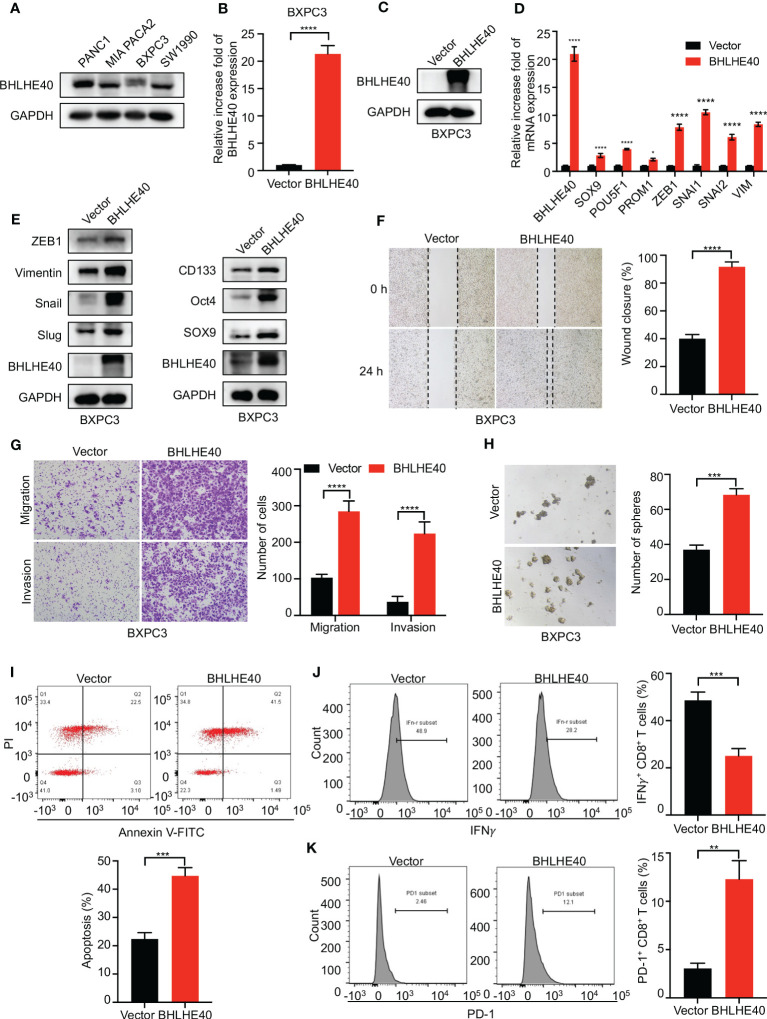
Functional assessment of BHLHE40 *in vitro*. **(A)** BHLHE40 expression in PDAC cell lines. **(B, C)** Validation of stable cellular BHLHE40 overexpression via Western blotting and RT-PCR. **(D, E)** Overexpression of BHLHE40 resulted in increased expression of stemness-related markers and EMT-related markers. **(F, G)** Wound-healing and cell migration and invasion assays showed that BHLHE40 enhanced cell migration and invasion. **(H)** Sphere formation assays showed that BHLHE40 promoted the stemness of tumor cells. **(I–K)** Flow cytometry analysis of CD8^+^ T-cell apoptosis and phenotype. PDAC, pancreatic ductal adenocarcinoma; EMT, epithelial–mesenchymal transition. *p<0.05; **p<0.01; ***p<0.001; ****p<0.0001.

The function of BHLHE40 was investigated *in vitro*. BHLHE40 expression was assessed in several PDAC cell lines, including PANC1, MIA PACA2, BXPC3, and SW1990. Of these, expression was the lowest in BXPC3 cells (**Figure 9A**). Lentivirus was then used to construct BHLHE40-overexpressing BXPC3 cell lines, and stable expression was validated at the mRNA level and the protein level (**Figures 9B, C**). Consistent with previous results, BHLHE40 overexpression resulted in increased expression of stemness-related markers (SOX9, Oct4, and CD133) and EMT-related markers (ZEB1, Snail, Slug, and Vimentin) at both the mRNA level and the protein level (**Figures 9D, E**). EMT is closely associated with tumor invasion and metastasis, and in the current study, BHLHE40 overexpression promoted cell migration and invasion (**Figures 9F, G**). BHLHE40 overexpression increased the sphere formation capacity of tumor cells (**Figure 9H**). To further investigate the role of BHLHE40 in the tumor immune microenvironment, CD8^+^ T cells were extracted from human peripheral blood and cocultured with tumor cells. When cocultured with tumor cells overexpressing BHLHE40, CD8^+^ T-cell apoptosis was increased (**Figure 9I**), the number of IFNγ^+^CD8^+^ T cells was significantly decreased, and the number of PD-1^+^CD8^+^ T cells was significantly increased (**Figures 9J, K**). These observations indicated that BHLHE40 could promote CD8^+^ T-cell apoptosis and inhibit the anti-tumor capacity of T cells. They also suggested that BHLHE40 played a critical role in the TIME, promoting the development of pancreatic cancer.

## 4 Discussion

Pancreatic cancer, especially PDAC, has attracted much attention over the years because it is highly invasive, metastatic, and incurable (22). Most patients with pancreatic cancer received treatment in advanced stages and have lost the opportunity for surgical treatment (23). It is therefore necessary to improve the detection of early-stage pancreatic cancer (23, 24). Hence, we designed the current study to identify biomarkers suitable for early screening and prognosis prediction.

In the present study, transcriptome sequencing was performed in 10 patients with PDAC, and immune infiltration analysis was conducted using CIBERSORT and ESTIMATE algorithms. Reduced immune cell infiltration, tumor stemness, and dense stroma make PDAC a “cold” tumor, resulting in insensitivity to radiotherapy, chemotherapy, and immunotherapy (25–27). The 10 patients were divided into high and low groups based on CD8^+^ T-cell infiltration ratio, CAF proportions, and tumor stemness index scores, and DEG analysis was performed. The results indicated that patients with higher stromal scores, higher tumor stemness index scores, and lower immune scores may suffer from more aggressive tumors and worse outcomes. Lastly, 40 expression-enriched molecules involved in important processes such as the NF-κB signaling pathway, cell adhesion, and MAPK signaling were screened out via intersection analysis.

Next, TCGA database analysis and LASSO regression analysis were used to identify four potential biological molecules (ITGA2, ITGA3, BHLHE40, and ADAM9) that could significantly affect the prognosis of PDAC patients. ITGA2 and ITGA3 are members of the integrin-encoding α chain protein family, which bind to the β subunit and participate in extracellular matrix adhesion (28). It has recently been reported that ITGA2 promotes the invasion of malignant tumors by activating the STAT3 signaling pathway and subsequently upregulating PD-L1 expression (29). In other studies, ITGA2 was highly expressed in PDAC tumor tissue, and ITGA2 expression was significantly associated with gemcitabine resistance and poor prognoses in PDAC patients (30, 31). ITGA3 evidently promotes extracellular matrix remodeling and metastasis in pancreatic cancer (32), its expression is increased in a variety of tumors, and these events are closely related to tumor metastasis and poor prognoses (33, 34). BHLHE40 is a helix-loop-helix-type transcription factor that plays a critical role in cell differentiation and proliferation (35), and in many studies, BHLHE40 has been closely associated with the regulation of the TIME and cancer progression (16, 36–38). ADAM9 is a disintegrin and a metalloprotease, and ADAM9 overexpression can result in a poor outcome via various biological processes such as the promotion of angiogenesis, cell migration, and invasion (39–41). The present study indicated that ITGA2, ITGA3, ADAM9, and BHLHE40 were potential target genes associated with the development of pancreatic cancer.

The widespread use of single-cell sequencing has improved the understanding of the composition and dynamic evolution of TME (42). In single-cell sequencing analysis, ITGA2, ITGA3, ADAM9, and BHLHE40 were all highly expressed in malignant ductal cells, suggesting important roles in tumor progression. ITGA2, ITGA3, ADAM9, and BHLHE40 were also of strong diagnostic value in ROC curve analyses. Patients with high expression of ITGA2, ITGA3, ADAM9, and BHLHE40 had shorter overall survival times, higher differential grades, higher pathologic staging, and worse therapy outcomes, suggesting that these biomarkers were predictive with respect to PDAC development. Lastly, a combination of these four molecules was used to construct a risk model and confirm that the risk model could effectively predict patient prognoses and survival times.

To confirm our findings, we also performed WGCNA to screen for critical biological molecules associated with pancreatic cancer development. BHLHE40 was identified as one of a number of core genes involved in the development of pancreatic cancer. It has been reported that BHLHE40 can promote SNAI1 and SNAI2 expression and inhibit the expression of CLDN1, CLDN4, and CDH2 in breast cancer, promoting the migration and invasion of tumor cells (43). BHLHE40 is also an important immune regulatory factor that can inhibit anti-tumor immunity by affecting the levels of cytokines secreted by T cells (38). In the current study, BHLHE40 expression was negatively associated with many different types of T cells and suppression of anti-cancer immune responses. PSCs are closely associated with pancreatic fibrosis and EMT during cancer progression, and BHLHE40 was positively correlated with genes associated with PSC activation, the EMT process, and cancer cell stemness formation, suggesting that high BHLHE40 expression could promote interstitial fibrosis and pancreatic cancer progression. Notably, enrichment analysis suggested that BHLHE40 could activate vital biological pathways such as NF-κB and MAPK signals. These observations require further verification. Clinical sample analysis further validated the important role of BHLHE40 in the prognosis and progression of PDAC, and BHLHE40 was positively correlated with lymph node metastasis, T stage, and AJCC stage and negatively correlated with survival time. *In vitro* experiments confirmed the function of BHLHE40, with respect to promoting cell migration and invasion via the EMT process, enhancing the stemness of tumor cells, and inhibiting anti-tumor immunity via the promotion of CD8^+^ T-cell apoptosis.

The current study had some limitations. It was necessary to characterize an updated long-term clinical validation cohort from our research center. Although TCGA-PAAD database has the advantage of sufficient sample size, including 189 samples from predominantly white pancreatic cancer patients, PDAC samples constitute the majority of TCGA-PAAD database samples (n = 150), and other non-PDAC samples are represented in lower numbers or not represented at all, such as PNTE. Therefore, TCGA-PAAD database has the limitation of race bias and uneven distribution of samples with different pathological types, and there will be a certain degree of bias in the analysis. Notably, however, this does not compromise our conclusion that BHLHE40 plays a crucial role in PDAC. Second, in order to confer clinical transitional significance upon BHLHE40, further comprehensive *in vivo* and *in vitro* investigations are warranted to elucidate the precise molecular mechanisms underlying its involvement in PDAC development, including the exploration of BHLHE40 inhibitors combined with chemotherapy or immunotherapy.

In summary, the present study identified core genes associated with immunity, stroma, and tumor stemness in pancreatic cancer and established a prognostic model that may facilitate better screening of high-risk groups and more accurate determination of PDAC prognosis. BHLHE40 was the most promising molecule in the model. It was significantly associated with PDAC prognosis, induction of an immunosuppressive microenvironment, and the promotion of cancer metastasis.

## Data availability statement

The datasets presented in this study can be found in online repositories. The names of the repository/repositories and accession number(s) can be found in the article/**Supplementary Material**.

## Ethics statement

This study was approved by the ethics committee of Tianjin Medical University Cancer Hospital and all patients provided written informed consent.

## Author contributions

CL, JD and JWZ designed the study, wrote the manuscript, and performed the experiments. RZ, JLZ, BX and LD analyzed and visualized the data. QZ, XY and SG revised the manuscript. YR, YW and XZ provided guidance and reviewed the paper. All authors contributed to the article and approved the submitted version.

## Supplementary material

Supplementary Figure 1 K-M survival curves of ITGA2, ITGA3, ADAM9, and BHLHE40. **(A)**. Associations between overall survival and the expression of ITGA2, ITGA3, ADAM9, and BHLHE40. **(B)**. Associations between disease-free survival and the expression of ITGA2, ITGA3, ADAM9, and BHLHE40.

Supplementary Figure 2 Single-cell analysis. **(A)**. Identification of malignant ductal cells with KRT19, EPCAM, MUC1, and FXYD3. **(B)**. Violin plot of ITGA2, ITGA3, ADAM9, and BHLHE40.

Supplementary Figure 3 Clinical correlation analysis. Associations between ITGA2, ITGA3, ADAM9, and BHLHE40 and T stage and primary therapy outcome.

## References

[1] Park W Chawla A O'reilly EM. Pancreatic cancer: a review. Jama (2021) 326:851–62. doi: 10.1001/jama.2021.13027 PMC9363152 34547082

[2] Kamisawa T Wood LD Itoi T Takaori K. Pancreatic cancer. Lancet (2016) 388:73–85. doi: 10.1016/S0140-6736(16)00141-0 26830752

[3] Siegel RL Miller KD Fuchs HE Jemal A. Cancer statistic. CA Cancer J Clin (2021) 71:7–33. doi: 10.3322/caac.21654 33433946

[4] Fang YT Yang WW Niu YR Sun YK. Recent advances in targeted therapy for pancreatic adenocarcinoma. World J Gastrointest Oncol (2023) 15:571–95. doi: 10.4251/wjgo.v15.i4.571 PMC10134207 37123059

[5] Zuo D Chen Y Zhang X Wang Z Jiang W Tang F. Identification of hub genes and their novel diagnostic and prognostic significance in pancreatic adenocarcinoma. Cancer Biol Med (2021) 19:1029–46. doi: 10.20892/j.issn.2095-3941.2020.0516 PMC9334760 34403221

[6] Jones S Zhang X Parsons DW Lin JC Leary RJ Angenendt P. Core signaling pathways in human pancreatic cancers revealed by global genomic analyses. Science (2008) 321:1801–6. doi: 10.1126/science.1164368 PMC2848990 18772397

[7] Hinshaw DC Shevde LA. The tumor microenvironment innately modulates cancer progression. Cancer Res (2019) 79:4557–66. doi: 10.1158/0008-5472.CAN-18-3962 PMC6744958 31350295

[8] Ren B Cui M Yang G Wang H Feng M You L. Tumor microenvironment participates in metastasis of pancreatic cancer. Mol Cancer (2018) 17:108. doi: 10.1186/s12943-018-0858-1 30060755 PMC6065152

[9] Bulle A Lim KH. Beyond just a tight fortress: contribution of stroma to epithelial-mesenchymal transition in pancreatic cancer. Signal Transduct Target Ther (2020) 5:249. doi: 10.1038/s41392-020-00341-1 33122631 PMC7596088

[10] Hessmann E Buchholz SM Demir IE Singh SK Gress TM Ellenrieder V. Microenvironmental determinants of pancreatic cancer. Physiol Rev (2020) 100:1707–51. doi: 10.1152/physrev.00042.2019 32297835

[11] Ho WJ Jaffee EM Zheng L. The tumour microenvironment in pancreatic cancer - clinical challenges and opportunities. Nat Rev Clin Oncol (2020) 17:527–40. doi: 10.1038/s41571-020-0363-5 PMC7442729 32398706

[12] Nallasamy P Nimmakayala RK Karmakar S Leon F Seshacharyulu P Lakshmanan I. Pancreatic tumor microenvironment factor promotes cancer stemness via SPP1-CD44 axis. Gastroenterology (2021) 161:1998–2013.e7. doi: 10.1053/j.gastro.2021.08.023 34418441 PMC10069715

[13] Chen Z Song H Zeng X Quan M Gao Y. Screening and discrimination of optimal prognostic genes for pancreatic cancer based on a prognostic prediction model. G3 (Bethesda) (2021) 11(11):jkab296. doi: 10.1093/g3journal/jkab296 34499727 PMC8527504

[14] Zou W Li L Wang Z Jiang N Wang F Hu M. Up-regulation of S100P predicts the poor long-term survival and construction of prognostic signature for survival and immunotherapy in patients with pancreatic cancer. Bioengineered (2021) 12:9006–20. doi: 10.1080/21655979.2021.1992331 PMC8806945 34654352

[15] Song J Ruze R Chen Y Xu R Yin X Wang C. Construction of a novel model based on cell-in-cell-related genes and validation of KRT7 as a biomarker for predicting survival and immune microenvironment in pancreatic cancer. BMC Cancer (2022) 22:894. doi: 10.1186/s12885-022-09983-6 35974300 PMC9380297

[16] Sethuraman A Brown M Krutilina R Wu ZH Seagroves TN Pfeffer LM. BHLHE40 confers a pro-survival and pro-metastatic phenotype to breast cancer cells by modulating HBEGF secretion. Breast Cancer Res (2018) 20:117. doi: 10.1186/s13058-018-1046-3 30285805 PMC6167787

[17] Jia Y Liu Y Zhu J Liu L Ma X Liu D. DEC1 promotes progression of helicobacter pylori-positive gastric cancer by regulating Akt/NF-κB pathway. J Cell Mol Med (2022) 26:1943–54. doi: 10.1111/jcmm.17219 PMC8980912 35122398

[18] Sui Y Jiang H Kellogg CM Oh S Janknecht R. Promotion of colorectal cancer by transcription factor BHLHE40 involves upregulation of ADAM19 and KLF7. Front Oncol (2023) 13:1122238. doi: 10.3389/fonc.2023.1122238 36890812 PMC9986587

[19] Yuan NL Liu Y Zhang D. Role of differentiated embryo-chondrocyte expressed gene 1 (DEC1) in immunity. Int Immunopharmacol (2022) 102:107892. doi: 10.1016/j.intimp.2021.107892 34215553

[20] Langfelder P Horvath S. WGCNA: an r package for weighted correlation network analysis. BMC Bioinf (2008) 9:559. doi: 10.1186/1471-2105-9-559 PMC2631488 19114008

[21] Bindea G Mlecnik B Tosolini M Kirilovsky A Waldner M Obenauf AC. Spatiotemporal dynamics of intratumoral immune cells reveal the immune landscape in human cancer. Immunity (2013) 39:782–95. doi: 10.1016/j.immuni.2013.10.003 24138885

[22] Vincent A Herman J Schulick R Hruban RH Goggins M. Pancreatic cancer. Lancet (2011) 378:607–20. doi: 10.1016/S0140-6736(10)62307-0 PMC3062508 21620466

[23] Zhang L Sanagapalli S Stoita A. Challenges in diagnosis of pancreatic cancer. World J Gastroenterol (2018) 24:2047–60. doi: 10.3748/wjg.v24.i19.2047 PMC5960811 29785074

[24] Tonini V Zanni M. Early diagnosis of pancreatic cancer: what strategies to avoid a foretold catastrophe. World J Gastroenterol (2022) 28:4235–48. doi: 10.3748/wjg.v28.i31.4235 PMC9453775 36159004

[25] Valle S Alcalá S Martin-Hijano L Cabezas-Sáinz P Navarro D Muñoz ER. Exploiting oxidative phosphorylation to promote the stem and immunoevasive properties of pancreatic cancer stem cells. Nat Commun (2020) 11:5265. doi: 10.1038/s41467-020-18954-z 33067432 PMC7567808

[26] Ullman NA Burchard PR Dunne RF Linehan DC. Immunologic strategies in pancreatic cancer: making cold tumors hot. J Clin Oncol (2022) 40:2789–805. doi: 10.1200/JCO.21.02616 PMC9390820 35839445

[27] Wang D Li Y Ge H Ghadban T Reeh M Güngör C. The extracellular matrix: a key accomplice of cancer stem cell migration, metastasis formation, and drug resistance in PDAC. Cancers (Basel) (2022) 14:16. doi: 10.3390/cancers14163998 PMC9406497 36010993

[28] Desgrosellier JS Cheresh DA. Integrins in cancer: biological implications and therapeutic opportunities. Nat Rev Cancer (2010) 10:9–22. doi: 10.1038/nrc2748 20029421 PMC4383089

[29] Ren D Zhao J Sun Y Li D Meng Z Wang B. Overexpressed ITGA2 promotes malignant tumor aggression by up-regulating PD-L1 expression through the activation of the STAT3 signaling pathway. J Exp Clin Cancer Res (2019) 38:485. doi: 10.1186/s13046-019-1496-1 31818309 PMC6902401

[30] Deichmann S Schindel L Braun R Bolm L Taylor M Deshpande V. Overexpression of integrin alpha 2 (ITGA2) correlates with poor survival in patients with pancreatic ductal adenocarcinoma. J Clin Pathol (2022). doi: 10.1136/jclinpath-2022-208176 35396216

[31] Gregori A Bergonzini C Capula M Mantini G Khojasteh-Leylakoohi F Comandatore A. Prognostic significance of integrin subunit alpha 2 (ITGA2) and role of mechanical cues in resistance to gemcitabine in pancreatic ductal adenocarcinoma (PDAC). Cancers (Basel) (2023) 15:3. doi: 10.3390/cancers15030628 PMC9913151 36765586

[32] Liu M Zhang Y Yang J Zhan H Zhou Z Jiang Y. Zinc-dependent regulation of ZEB1 and YAP1 coactivation promotes epithelial-mesenchymal transition plasticity and metastasis in pancreatic cancer. Gastroenterology (2021) 160:1771–1783.e1. doi: 10.1053/j.gastro.2020.12.077 33421513 PMC8035249

[33] Tang XR Wen X He QM Li YQ Ren XY Yang XJ. MicroRNA-101 inhibits invasion and angiogenesis through targeting ITGA3 and its systemic delivery inhibits lung metastasis in nasopharyngeal carcinoma. Cell Death Dis (2017) 8:e2566. doi: 10.1038/cddis.2016.486 28102841 PMC5386386

[34] Zhang G Li B Lin Y. Evaluation of ITGA3 as a biomarker of progression and recurrence in papillary thyroid carcinoma. Front Oncol (2021) 11:614955. doi: 10.3389/fonc.2021.614955 35174063 PMC8841514

[35] Asanoma K Liu G Yamane T Miyanari Y Takao T Yagi H. Regulation of the mechanism of TWIST1 transcription by BHLHE40 and BHLHE41 in cancer cells. Mol Cell Biol (2015) 35:4096–109. doi: 10.1128/MCB.00678-15 PMC4648814 26391953

[36] Zhang L Yu X Zheng L Zhang Y Li Y Fang Q. Lineage tracking reveals dynamic relationships of T cells in colorectal cancer. Nature (2018) 564:268–72. doi: 10.1038/s41586-018-0694-x 30479382

[37] Li C Zhu B Son YM Wang Z Jiang L Xiang M. The transcription factor Bhlhe40 programs mitochondrial regulation of resident CD8(+) T cell fitness and functionality. Immunity (2019) 51:491–507.e7. doi: 10.1016/j.immuni.2019.08.013 31533057 PMC6903704

[38] Cook ME Jarjour NN Lin CC Edelson BT. Transcription factor Bhlhe40 in immunity and autoimmunity. Trends Immunol (2020) 41:1023–36. doi: 10.1016/j.it.2020.09.002 PMC7606821 33039338

[39] Oria VO Lopatta P Schmitz T Preca BT Nyström A Conrad C. ADAM9 contributes to vascular invasion in pancreatic ductal adenocarcinoma. Mol Oncol (2019) 13:456–79. doi: 10.1002/1878-0261.12426 PMC6360373 30556643

[40] Chou CW Huang YK Kuo TT Liu JP Sher YP. An overview of ADAM9: structure, activation, and regulation in human diseases. Int J Mol Sci (2020) 21:20. doi: 10.3390/ijms21207790 PMC7590139 33096780

[41] Lin YS Kuo TT Lo CC Cheng WC Chang WC Tseng GC. ADAM9 functions as a transcriptional regulator to drive angiogenesis in esophageal squamous cell carcinoma. Int J Biol Sci (2021) 17:3898–910. doi: 10.7150/ijbs.65488 PMC8495400 34671207

[42] Lei Y Tang R Xu J Wang W Zhang B Liu J. Applications of single-cell sequencing in cancer research: progress and perspectives. J Hematol Oncol (2021) 14:91. doi: 10.1186/s13045-021-01105-2 34108022 PMC8190846

[43] Zheng Q Wang C Wang L Zhang D Liu N Ming X. Interaction with SP1, but not binding to the e-box motifs, is responsible for BHLHE40/DEC1-induced transcriptional suppression of CLDN1 and cell invasion in MCF-7 cells. Mol Carcinog (2018) 57:1116–29. doi: 10.1002/mc.22829 29704436

