# Supplementary figures and images for: The role of BHLHE40 in clinical features and prognosis value of PDAC by comprehensive analysis and in vitro validation
Source: Front Oncol. 2023 Jun 12;13:1151321. doi: 10.3389/fonc.2023.1151321 (PMC10291124; doi:10.3389/fonc.2023.1151321)

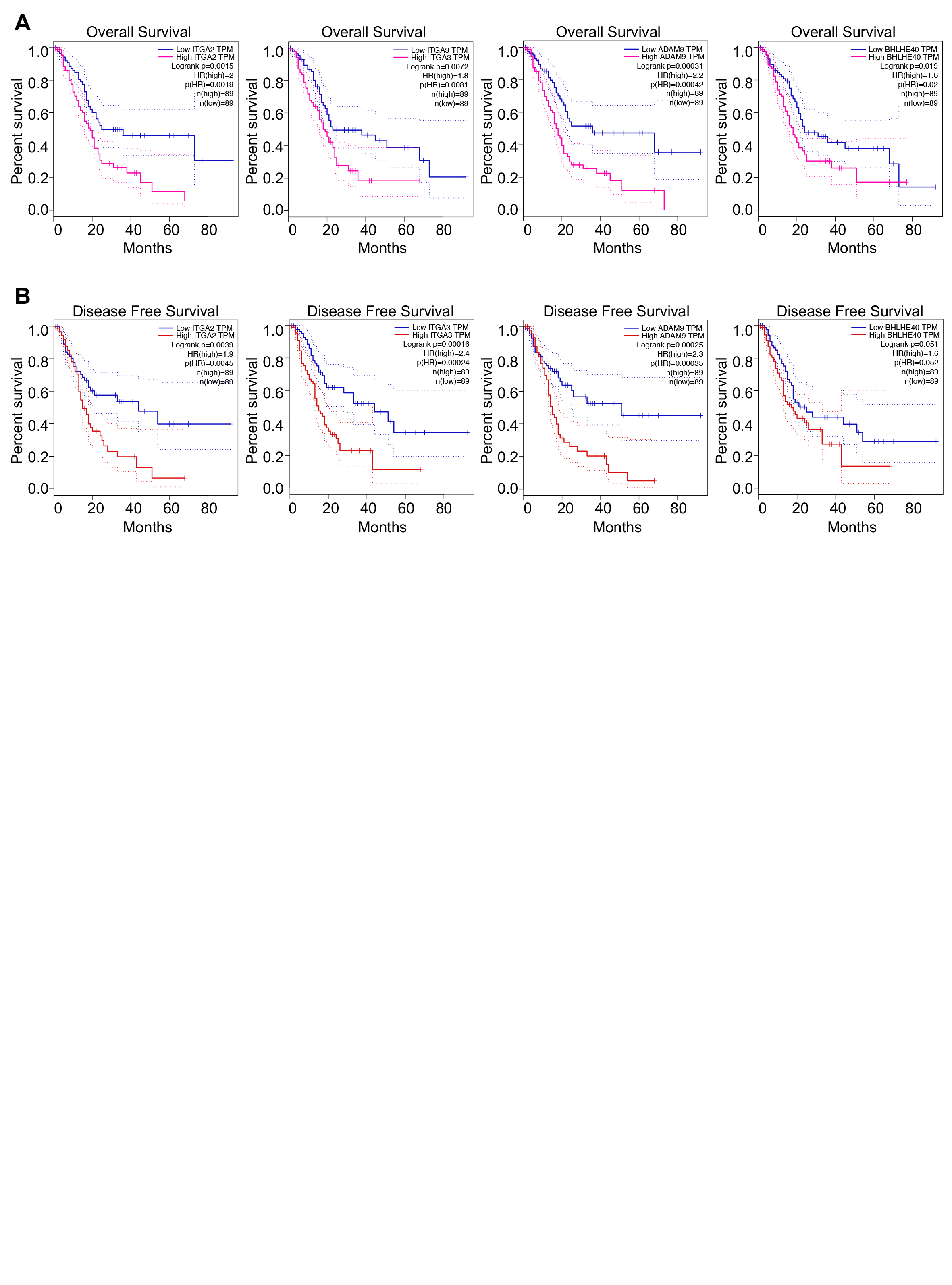

Supplement: Supplementary Figure 1 — K-M survival curves of ITGA2, ITGA3, ADAM9, and BHLHE40. (A). Associations between overall survival and the expression of ITGA2, ITGA3, ADAM9, and BHLHE40. (B). Associations between disease-free survival and the expression of ITGA2, ITGA3, ADAM9, and BHLHE40. [file Image_1.tif]

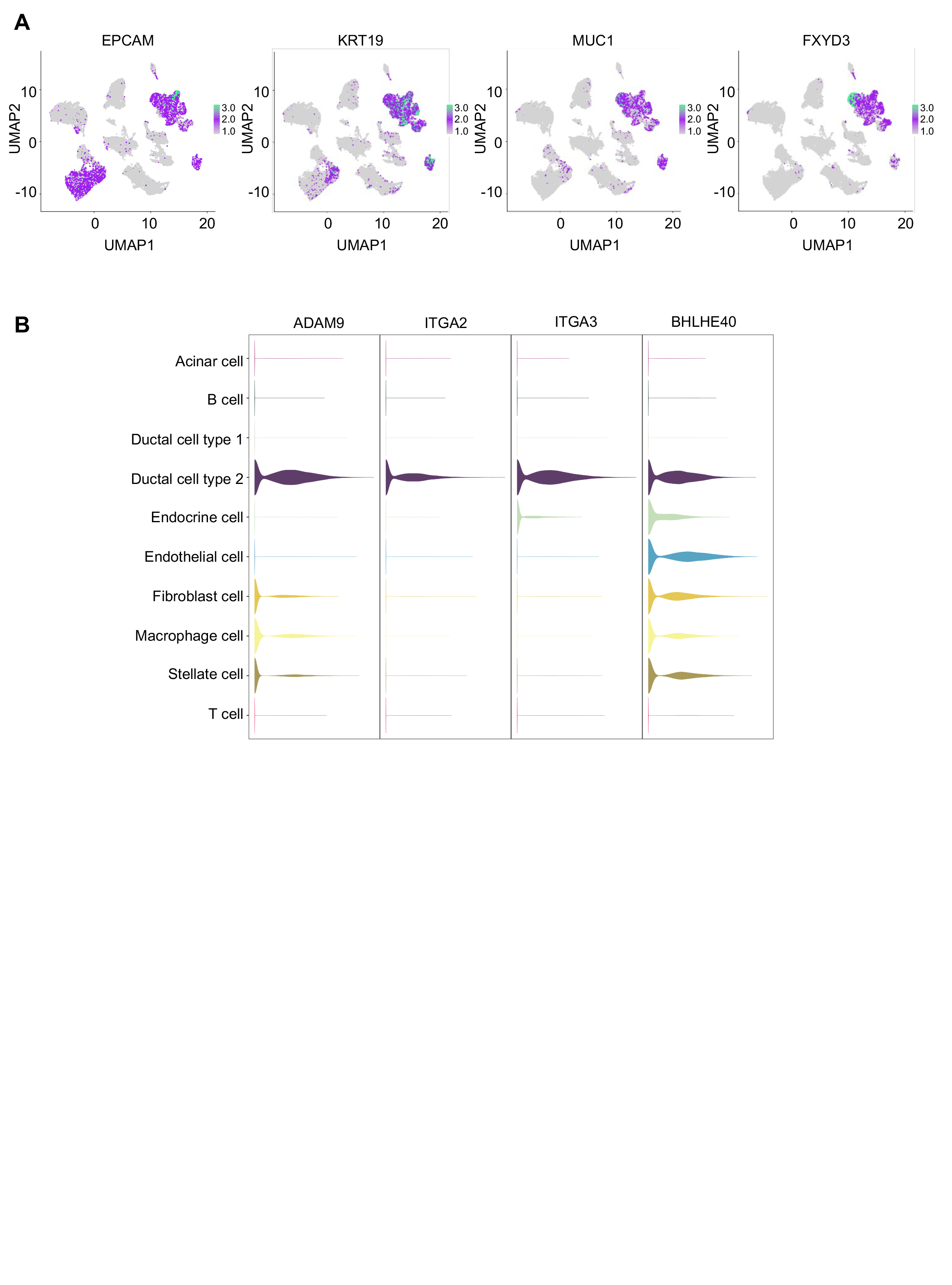

Supplement: Supplementary Figure 2 — Single-cell analysis. (A). Identification of malignant ductal cells with KRT19, EPCAM, MUC1, and FXYD3. (B). Violin plot of ITGA2, ITGA3, ADAM9, and BHLHE40. [file Image_2.tif]

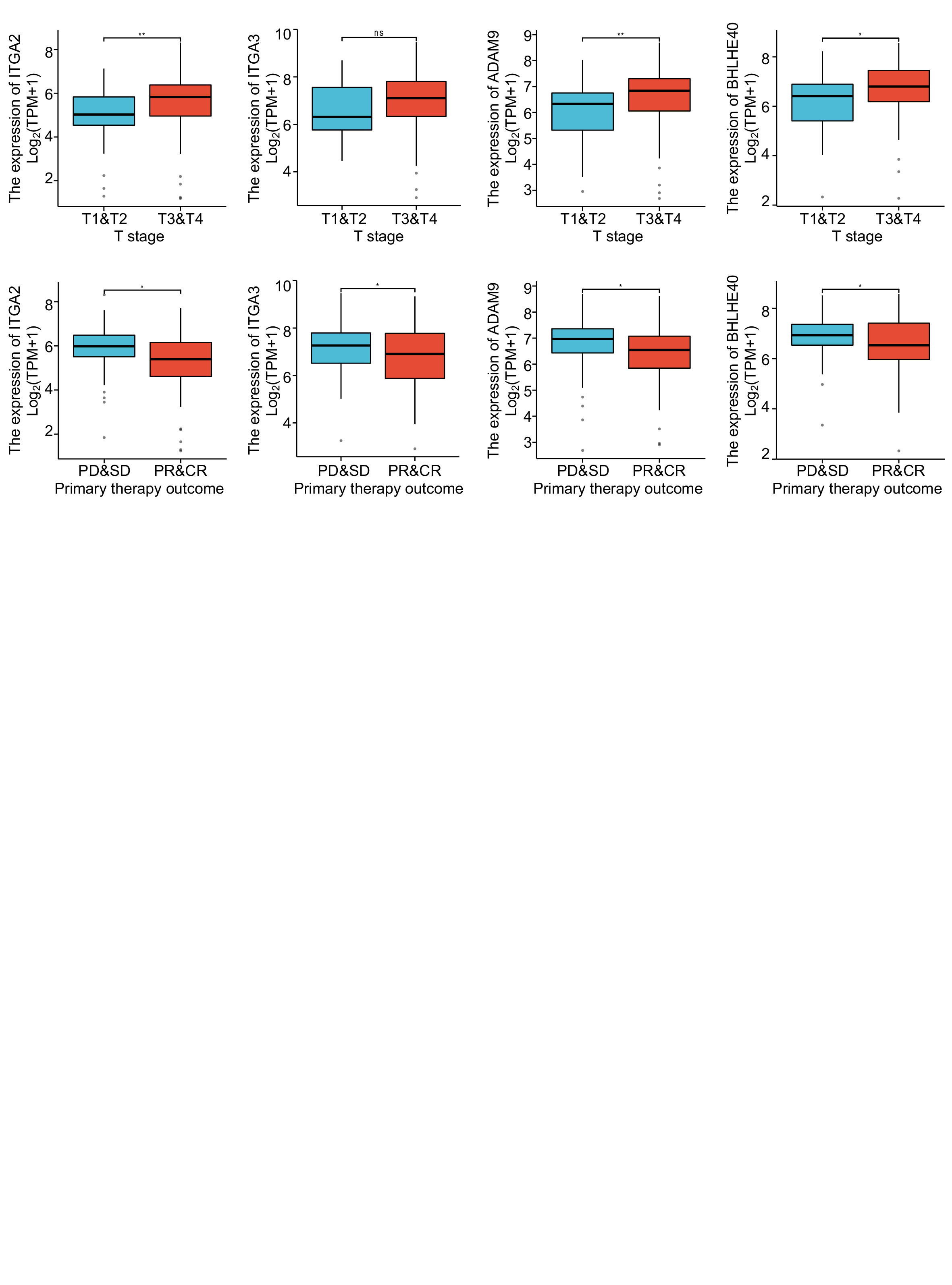

Supplement: Supplementary Figure 3 — Clinical correlation analysis. Associations between ITGA2, ITGA3, ADAM9, and BHLHE40 and T stage and primary therapy outcome. [file Image_3.tif]
